# Community Pharmacists role in obesity treatment in Kuwait: a cross-sectional study

**DOI:** 10.1186/1471-2458-12-863

**Published:** 2012-10-11

**Authors:** Abdelmoneim Awad, Mohammad Waheedi

**Affiliations:** 1 Department of Pharmacy Practice, Faculty of Pharmacy, Kuwait University, Kuwait, Kuwait

**Keywords:** Community pharmacists, Obesity, Obesity counseling, Kuwait

## Abstract

**Background:**

Obesity is a growing health concern in Kuwait. Obesity has been identified as a key risk factor for many chronic diseases including hypertension, dyslipidemia and type 2 diabetes mellitus. It has been shown that community pharmacists' involvement is associated with successful weight management in developed countries. This study was conducted to investigate the role of community pharmacists in obesity counseling, and to identify the barriers to counseling in Kuwait.

**Methods:**

A descriptive cross-sectional study involved 220 community pharmacies that were selected via stratified and systematic random sampling. A pretested self-administered questionnaire collected information on frequency and comfort level with obesity counseling, and the perceived effectiveness of four aspects of obesity management (diet and exercise, prescribed antiobesity medications, diet foods, and nonprescription products and dietary supplements). Information on perceived confidence in achieving positive outcomes as a result of counseling and barriers to counseling was also collected. Descriptive and Spearman’ r analysis were conducted using SPSS version 17. Responses with Likert scale rating 1(low score) to 5 (high score) and binary choices (yes/no) were presented as mean (SD) and (95% CI), respectively.

**Results:**

The response rate was 93.6%. The overall mean (SD) responses indicated that pharmacists counseled obese patients sometimes to most of the time, 3.67 (1.19) and were neutral to comfortable with counseling about aspects of obesity management, 3.77 (1.19). Respondents perceived obesity management aspects to be somewhat effective, 3.80 (1.05). Of the four aspects of obesity management, diet and exercise, and diet foods were the highest ranked in terms of frequency of counseling, comfort level and perceived effectiveness. Pharmacists were neutral to confident in achieving positive outcomes as a result of obesity counseling, 3.44 (1.09). Overall mean responses of counseling obese patients by pharmacists were positively correlated with their perceived comfort with counseling and perceived effectiveness of obesity management aspects. The most anticipated barriers to obesity counseling were lack of patient awareness about pharmacists' expertise in counseling 76.2% (95% CI: 69.7-81.7) and pharmacists’ opinions that obese patients lack willpower and are non-adherent to weight reduction interventions 71.8% (95% CI: 65.1-77.8).

**Conclusions:**

Strengths, weaknesses and barriers related to obesity counseling by pharmacists in Kuwait were identified, and suggestions were provided to strengthen that role.

## Background

Obesity and overweight represent a rapidly growing public health challenge worldwide. World Health Organization (WHO) estimates that 1.5 billion adults and about 43 million children under 5 years old were overweight in 2010. In general, greater than one in ten of the world’s adult individuals are obese [1]. Obesity and overweight are the fifth leading risk for worldwide deaths [1]. Obesity is a risk factor for development of various diseases including coronary heart disease, dyslipidaemia, hypertension, stroke, some forms of cancer, type 2 diabetes mellitus, gallbladder disease, osteoarthritis and gout, and pulmonary diseases, including sleep apnoea [2].

In Kuwait, obesity is a growing health concern; several studies have shown the high prevalence of overweight and obesity among the population. In 2010, about 80% of women were overweight or obese, and childhood obesity continues to increase as well [3-6]. The most common factors contributing to overweight and obesity are overeating unhealthy high-fat foods and sedentary lifestyles [6]. Thus, there is an urgent need for the implementation of effective preventive measures for controlling the prevalence of obesity including public education about lifestyle modifications.

Barriers to the assessment and management of obesity by physicians include inadequate training, lack of time, lack of reimbursement, lack of access to dietician and uncertainty in relation to the efficacy of weight loss interventions [7]. Furthermore, the provision of diet sheets and referral to a dietician or a psychotherapist by physicians was found to be unsatisfactory by the obese patients [8]. In 1989, the definition of health promotion was expanded to emphasise the importance of cooperation between different stakeholders to enhance awareness, change behaviour and create environments that support good health practices [9]. Among them, community pharmacists have a valuable role in promoting and maintaining public health as one of the most accessible health care providers. Pharmacists have already started working with other health care professionals in the USA to maintain and promote public health [10]. A study conducted across Europe shows that 64% out of a total of over 6,000 people in six countries supported the further development of community pharmacies as alternatives to physicians' clinics, so that they can have more choice in receiving advice and treatment for common conditions. The greatest support for extending pharmacy based health care was in the United Kingdom (UK) and Poland [11].

Health promotion plans focus on community based interventions and partnerships to maintain wellness and to help modify individual behaviours such as unhealthy lifestyles [12]. In the UK and Australia, community pharmacies are recognised as the most accessible healthcare settings due to the high volume of the population that use their services [13]. Given the numerous comorbidities characteristically exist with obesity, obese patients often interact with their community pharmacists to obtain prescription drugs and/or over-the-counter (OTC) products for these diseases. In fact, pharmacist is the most accessible health care professional in the community who can help patients in the management of overweight and obesity by assisting in the selection of weight loss agents, offer appropriate counseling on nutritional and lifestyle modifications, and monitoring to ensure safe and effective pharmacotherapy outcomes [14-17]. A systematic review of community pharmacy-based weight management interventions reported the findings of 10 relevant studies conducted in the USA, the UK, Switzerland, Spain and Denmark [18]. In England, pharmacist-led weight management program has achieved significant results, and its success was one of the major driving forces behind the Department of Health’s recommendation that community pharmacists should be involved in weight management services [19].

Pharmacy associations, such as the American Society of Health-System Pharmacists encourages pharmacists to work with obese clients to reinforce lifestyle modifications and provide education and encouragement [20]. A number of the pharmacy profession roles were identified in a clinical guideline on the prevention and management of obesity published by the National Institute for Health and Clinical Excellence [21]. In developed countries, community pharmacists have been identified as health advisors and highly credible source of health information to the general public through their counseling activities, including helping clients achieve and maintain a healthy weight [18,19,22-26].

In a country like Kuwait, where overweight and obesity are prevalent, the contribution of all health care professionals including pharmacists in fighting this epidemic is crucial. Most studies on the pharmacists’ role in obesity counseling have been conducted in developed countries, and there is a lack of information on this role in the Middle East region including Kuwait. Therefore, the present study was designed to describe the self-reported practice of community pharmacists in relation to counseling patients with obesity, identify the practice barriers to obesity counseling as well as strategies to overcome barriers to counseling.

## Methods

A descriptive, cross-sectional survey was conducted in Kuwait. Kuwait is a Middle Eastern country with an area of 17.820 square kilometres and an estimated population 3.328.136 people (2007 estimate). The study population included full-licensed pharmacists working at community pharmacies in Kuwait. The ethical clearance for this study was obtained from the "Human Ethical Committee, Health Sciences Center, Kuwait University".

The sample size was determined using Java Applets for Power and Sample Size [27]. It was calculated that a sample size of 186 pharmacists would be required to determine a 20% difference in proportion between two groups (e.g., male vs. female) with an 80% power and a 5% significance level. The community pharmacies’ lists at different governorates were obtained from the Ministry of Health (current at the time of the study) for sample selection, due to the lack of lists with the names and addresses of community pharmacists in Kuwait. A larger sample of 220 community pharmacies (out of a total of 287) was selected to adjust for possible nonresponse, using stratified and systematic random sampling according to the methodology described by World Health Organization [28]. The stratification was at the level of the five governorates of Kuwait (Capital, Al-Ahmadi, Al-Farwaniyah, Hawalli and Al-Jahra). A pharmacy employing more than one registered pharmacist, only one was included.

The basis of developing the study questionnaire was obtained from validated surveys that investigated community pharmacists’ counseling on obesity management and barriers to obesity counseling in the USA [29,30]. The original surveys remained unchanged except for few items that were not applicable to pharmacists in Kuwait. The questionnaire contained both closed and open-ended questions. It was pretested for content, design, readability, and comprehension on 15 community pharmacists and modifications were made as necessary so that the questionnaire was simple to answer, yet gave accurate data. Demographic and other characteristics data collected included gender, age, basic and postgraduate qualifications, years practiced as a pharmacist and location of the pharmacy. Pharmacists were asked about how often they counseled patients regarding four aspects of obesity management: diet and exercise, prescribed antiobesity medications, diet foods, and nonprescription products and dietary supplements for weight loss. Responses were measured on a 5-point Likert scale (never [1], rarely [2], sometimes [3], most of the time [4], always [5]). Pharmacists' comfort level with counseling about the four aspects of obesity management was measured by a 5-point Likert scale (extremely uncomfortable [1], uncomfortable [2], neither comfortable nor uncomfortable [3], comfortable [4] extremely comfortable [5]). A 5-point Likert scale (extremely ineffective [1], ineffective [2], neither effective nor ineffective [3], effective [4], extremely effective [5]) was used to measure the respondents’ perceived effectiveness of the four aspects of obesity management. The extent to which pharmacists perceived confident in achieving positive outcomes as a result of counseling obese patients was measured by listing seven obesity management related items and asking respondents to indicate on a 5-point scale (extremely unconfident [1], unconfident [2], neither confident nor unconfident [3], confident [4], extremely confident [5]). The barriers that can limit pharmacists’ participation in counseling obese patients was measured by listing ten barriers, and asking respondents to indicate yes or no on each barrier. A 4-point Likert scale (not at all [1]; not very much [2]; to some extent [3]; to a great extent [4]) was used to measure the opinions of the respondents in relation to eight strategies that can help to overcome the barriers.

On approaching a pharmacy, four data collectors (pharmacists) briefly explained the purpose of the study to the community pharmacist on duty (face-to face). Pharmacists were free to refuse to participate in the survey. Those who agreed to take part in the study were given the questionnaires and then were collected from them anonymously after being completed within 1–2 weeks. They were assured for confidentiality and gave written consent to participate in the study. Incentives were not offered for completion of the questionnaire.

### Statistical analysis

Data were entered into the Statistical Package for Social Sciences (SPSS, version 17) and descriptive analysis conducted. Pharmacists’ responses were presented as means Likert scale rating (standard deviations) and percentages (95% confidence intervals). The confidence intervals were computed using EpiCalc 2000 (Brixton Health website). Student *t* tests were used to evaluate the differences in means between two groups of the independent variables (gender: males vs. females, age: 20–40 years vs. 41–60 years and experience as practitioners: ≤ 10 years vs. > 10 years). Statistical correlational analysis (Spearman’s r) was used to measure the association between respondents’ frequency of counselling and their comfort level with counseling, frequency of counselling and perceived effectiveness of aspects of obesity management; and frequency of counselling and perceived confidence in achieving positive outcomes. P < 0.05 was considered statistically significant.

## Results

### Study population

Two hundred and twenty pharmacists were approached regarding study participation, 206 of whom agreed to participate (93.6%). The mean (SD) age and experience as practitioners of the study participants were 33.5 (6.5) years and 9.5 (6.0) years, respectively. Table 1 shows the characteristics of respondents.

**Table 1:** Characteristics of study participants (n=206)

	**Frequency**	**Percentage (%)**
**Gender**		
Male	114	55.3
Female	92	44.7
**Age (years)**		
20–40	178	86.4
41-60	28	13.6
**Basic qualification in pharmacy**		
B. Pharm	198	96.1
M. Pharm	5	2.4
Pharm D	3	1.5
**Postgraduate qualification(s) in pharmacy**		
Diploma	15	7.3
Master degree	9	4.4
**Experience as practitioners (Years)**		
≤ 10	150	72.8
> 10	56	27.2
**Location of pharmacy (Governorates)**		
Hawalli	82	39.8
Al-Farwaniyah	41	20.0
Al-Ahmadi	33	16.0
Capital	31	15.0
Al-Jahra	19	9.2

### Respondents’ perceptions regarding obesity counseling

Table 2 shows the study participants’ responses regarding the frequency, comfort and effectiveness of four aspects of obesity management. The overall mean (SD) responses indicated that the respondents counseled patients sometimes to most of the time, 3.67 (1.19) and were neutral to comfortable with counseling patients about aspects of obesity management, 3.77 (1.19). Pharmacists perceived obesity management aspects to be somewhat effective in weight loss, 3.80 (1.05). Of the four aspects of obesity management diet and exercise, and diet foods were the highest ranked in terms of frequency of counseling, comfort level and perceived effectiveness. Respondents were neutral to confident in achieving positive outcomes as a result of obesity counseling, 3.44 (1.09). Pharmacists were most confident in achieving positive outcomes as a result of counseling about exercise and diet (Table 3).

**Table 2:** Responses to the frequency, comfort and effectiveness of aspects of obesity management (n=206)

**Aspects of obesity Management**	*Frequency of counseling Mean (SD)	**ˆComfort level with counseling Mean (SD)**	**#Perceived Effectiveness Mean (SD)**
Diet and exercise	4.22 (1.06)	4.28 (1.19)	4.47 (0.83)
Diet foods	4.03 (0.99)	4.06 (0.98)	4.11 (0.94)
Prescribed anti-obesity medications	3.29 (1.23)	3.40 (1.05)	3.37 (0.65)
Non-prescribed anti-obesity products and dietary supplements for weight loss	3.14 (1.09)	3.36 (1.29)	3.23 (1.27)
**Overall Scale mean**	3.67 (1.19)	3.77 (1.19)	3.80 (1.05)

*: Measured on a 5-point Likert scale: 1, never; 2, rarely; 3, sometimes; 4, most of the time; 5, always.

ˆ: Measured on a 5-point Likert scale: 1, extremely uncomfortable; 2, uncomfortable; 3, neither comfortable nor uncomfortable; 4, comfortable; 5, extremely comfortable.

#: Measured on a 5-point Likert scale: 1, extremely ineffective; 2, ineffective; 3, neither effective nor ineffective; 4, effective.; 5, extremely effective.

**Table 3:** Responses on perceived confidence in achieving positive outcomes as a result of counseling patients with obesity (n = 206)

**Outcomes**	**Perceived confidence Mean (SD)**
Eating a calorie-controlled diet	3.75 (1.57)
Engagement in regular exercise	3.73 (1.27)
Patient adherence to nutritional advice	3.68 (1.27)
Proper use of nonprescription products and dietary supplements for weight loss	3.59 (1.24)
Minimization of adverse effects from anti-obesity medications	3.46 (1.37)
Patient adherence to anti-obesity medications	3.43 (1.35)
Proper handling of missed doses of anti-obesity medications	3.21 (1.21)
**Overall scale mean**	3.44 (1.09)

Measured on a 5-point Likert scale: 1, extremely unconfident; 2, unconfident; 3, neither confident nor unconfident; 4, confident; 5, extremely confident.

Respondents’ frequency of counseling was significantly and positively associated with their comfort level with counseling obese patients (r=0.80; p < 0.001). Higher levels of perceived effectiveness of obesity management aspects were also significantly associated with higher levels of counseling obese patients (r=0.61; p < 0.001). Pharmacists’ frequency of obesity counseling was not significantly associated with their perceived confidence in achieving positive outcomes as a result of counseling (r=0.06; p = 0.07). There were no significant differences among the respondents’ characteristics in relation to frequency of counseling, comfort level and perceived effectiveness of options of obesity management (p > 0.05). Females were more confident than males in achieving positive outcomes in relation to patient adherence to antiobesity medications (p = 0.003), nutritional advice (p < 0.001), engagement in regular exercise (p = 0.02) and eating a calorie-controlled diet (p=0.008).

### Barriers to obesity counseling

Pharmacists indicated that the most anticipated barriers to counseling obese patients were lack of patient awareness about pharmacists' expertise in counseling 76.2% (95% CI: 69.7-81.7), pharmacists’ opinions that obese patients lack willpower and are non-adherent to weight reduction interventions 71.8% (95% CI: 65.1-77.8), and lack of pharmacists’ time 69.4% (95% CI: 62.6-75.5) (Table 4).

**Table 4:** Anticipated barriers to counseling obese patients (n =206)

**Barriers**	**% (95%CI)**
Lack of patient awareness about pharmacists' expertise in counseling	76.2 ( 69.7-81.7)
Opinions about people with obesity (i.e., lack of willpower and do not adhere to the interventions to reduce their weight)	71.8 (65.1-77.8)
Lack of pharmacist time for counseling	69.4 (62.6-75.5)
Lack of pharmacists who are expertise in counseling	55.8 (48.8-62.7)
Lack of patient demand for counseling	53.4 (46.4-60.3)
Lack of privacy for counseling	52.4 (45.4-59.4)
Lack of pharmacist interest in counseling	49.5 (42.5-56.5)
Lack of repayment to the pharmacist for counseling	48.1 (41.1-55.1)
Belief that obesity is controllable without medications	45.1 (38.3-52.2)
Opinions about obesity as not a disease	40.5 (33.6-47.4)

### Strategies to overcome barriers to obesity counseling

Pharmacists believed that increased awareness among public about pharmacists’ ability to counsel about obesity, improvement of pharmacists’ knowledge about obesity and communication skills as the top three strategies to overcome barriers to counseling (Table 5).

**Table 5:** Responses on strategies to overcome barriers to counseling patients with obesity (n=206)

**Strategies to overcome barriers to counseling**	**Mean (SD)**
Increased public awareness about pharmacists' abilities to counsel about obesity	3.57 (0.98)
Improvement of pharmacists’ knowledge about obesity	3.51 (0.72)
Improvement of pharmacists’ communication skills	3.47 (0.68)
Increased staffing in pharmacy	3.36 (1.20)
Greater support from pharmacy management for counseling activities	3.33 (0.96)
Development of a positive outlook toward patients with obesity	3.29 (1.08)
Reimbursement (repayment/compensation) to the pharmacists for counseling	3.06 (1.06)
Establishment of private consultation areas in the pharmacy	2.98 (1.27)

Measured on 4 -point Likert scale: 1, not at all; 2, not very much; 3, to some extent; 4, to a great extent.

## Discussion

### Key findings

This is the first study to our knowledge to be conducted in Kuwait, and probably in the Middle East region to describe the practice of community pharmacists in obesity management, and to identify the barriers to obesity counseling as well as strategies to overcome them. Overall, community pharmacists in Kuwait sometimes to most of the time counseled obese patients and were neutral to comfortable with counseling about obesity treatment. They perceived obesity management options to be somewhat effective in bringing about weight loss, but were neutral to confident in achieving positive outcomes with counseling. Lack of patient awareness about pharmacists' expertise in counseling was perceived to be the major barrier limiting community pharmacists in obesity counseling.

### Frequency of counseling patients with obesity

The present results revealed that pharmacists sometimes to most of the time counseled patients about various aspects of obesity management. This is contrary to the practice in the USA, where pharmacists rarely to sometimes counseled obese patients [29]. The involvement of the respondents in obesity counseling is a positive finding that needs to be encouraged to provide the appropriate care to obese patients. It was reported that community pharmacists are well positioned to provide weight management advice to the public, and their involvement is associated with successful weight loss [23-26]. Respondents who claimed that they counseled most of the time could have meant that they mostly counseled when asked by patients for services with regard to weight management, while others may have been proactive and perhaps offered weight management services to patients. However, this was not measured in this study, but it is more likely to be reactive than proactive; since it was reported that the wide involvement of pharmacists in drug dispensing duties, lack of time and lack of patients’ trust in the pharmacist’s ability to provide good health as key barriers to their active involvement in health promotion [31].

The current results identified that the respondents’ frequency of counseling regarding each of the four management options of obesity was positively correlated with their comfort level and perceived effectiveness of the obesity interventions. The study participants counseled most frequently about diet and exercise and diet foods, but less frequently about prescription and non-prescription antiobesity products compared to the USA study, where prescribed antiobesity medications followed by diet and exercise were found to be the most frequent counseling interventions [29]. Similar findings were reported by a pharmacy survey in Europe that 90% of the pharmacists thought that guidance on healthy eating should be provided; whilst 74% thought that they should offer advice on drug therapy for weight loss [32].

### Comfort level with obesity counseling

Respondents indicated a high comfort level with delivering lifestyle counseling, which have been perceived by them as the most effective intervention. This a positive finding since lifestyle modification has been considered as the first-line treatment for weight reduction, and significantly proved to reduce the incidence of type 2 diabetes compared to drug therapy with metformin [33]. However, pharmacists are not dieticians, but they can be given specific training to enable them to provide appropriate dietary advice involving instruction on healthy eating. Evidence shows that providing behavioural support to people increases the chance of successful weight loss [34]. In Kuwait, pharmacists may also need to deliver weight management programs that include behaviour change strategies to increase individuals’s physical activity levels, improve eating behaviour and the quality of the individual’s diet. It is evident that these multi-component interventions are the treatment of choice [21].

### Perceived effectiveness of obesity treatments

Of interest that the study participants perceived OTC medications and dietary supplements as the least effective therapeutic modality. Despite the fact that many pharmacists promote the use of dietary supplements and herbals in their practice, some studies have shown that pharmacists perceive them relatively less effective compared to dietary changes and exercise for management of obesity and coronary heart disease [30,35]. There is currently insufficient scientific evidence to support the use of OTC remedies for weight loss and to suggest that they are safe. OTC products are potentially helpful although likely yield no significant long-term weight loss. They can have a very diverse unpredictable levels of ingredients, and may be dangerous to the consumers’ health due to the toxicity of herbs used and herb-drug interactions [36]. A serious problem with herbal antiobesity remedies is the possibility that these products contain adulterants in their ingredients like sibutramine and thyroid extracts, which may have potential negative drug-herb interactions [36]. Thus, pharmacists in Kuwait need to be more involved in offering professional opinion, counseling and/or monitoring obese patients who are taking these antiobesity OTC products.

### Confidence in achieving positive outcomes as a result of obesity counseling

The present findings revealed that community pharmacists’ overall response with respect to their confidence in achieving positive outcomes as a result of their counseling with obesity was neutral, which is similar to that reported by the USA study [29]. However, unlike pharmacists in the USA study, who were most confident about achieving positive medication-related outcomes, pharmacists in Kuwait were most confident about achieving positive lifestyle-related outcomes. The less involvement of the respondents in counseling about prescription and non-prescription antiobesity products may be explained by their neutral responses to comfort level and effectiveness of both therapeutic interventions. This may be due to their beliefs that these medications are less effective compared to diet and exercise, and with poor safety profiles. Furthermore, the low use of antiobesity therapies in Kuwait compared to other countries could be another contributing factor. In Kuwait, a study was conducted to investigate the general practitioners’ attitudes and current practices in obesity management showed that most of the respondents preferred not to prescribe antiobesity medications [37].

Since counseling is one of the important aspects of pharmaceutical care, our findings highlight the need for pharmacists to be always involved in counseling patients concerning prescribed antiobesity medications. They should have a responsibility to counsel the patient before dispensing the medication providing proper directions of use, advice on missed doses, side effects and storage. Appropriate pharmacists’ counseling on prescribed antiobesity medications was found to improve patients’ adherence and achieve weight loss [23].

### Barriers to obesity counseling

Lack of patient awareness about pharmacists' expertise in counseling, pharmacists’ opinions that obese patients lack willpower and non-adherent to weight reduction interventions, and lack of pharmacists’ time were identified as the main barriers to obesity counseling. It was reported that the public in Kuwait has a poor image of pharmacists due to the limited interactions between pharmacists and patients, which has resulted in lack of recognition for pharmacy services by patients [38]. It was found that patients will not accept care provided by pharmacists for many reasons such as their unfamiliarity with the pharmacists’ expertise, their concern about the value of this service, and their belief that pharmacists are invading on their physician’s territory [39]. This barrier can be overcome through the pharmacists’ beliefs in their knowledge and skills, and the effective provision of a pharmaceutical care practice with great confidence that will convince patients of its value [39]. Education of patients about the importance of health care, and the role of pharmacists in providing counseling about health promotion behaviours can help in increasing patient awareness about the care that pharmacists can provide. It is also crucial to generate the perception in the community that their local pharmacist is the logical first source of health advice who will become a trusted service provider to them individually.

The strategy that was perceived by respondents as having the greatest impact on overcoming barriers to obesity counseling was to create awareness among patients about pharmacists' ability to counsel about obesity. Additionally, pharmacists need to become better prepared for these new roles. This can be achieved by the improvement of pharmacists’ knowledge about obesity and communication skills as being indicated by respondents. Similar findings were reported by a survey conducted in six European countries that 96% of pharmacists believed that with support and training they are well placed to help persons with their weight loss efforts [11]. Hence, a joint sustained collaboration between the Ministry of Health, the Pharmaceutical and Medical Associations and Kuwait University is essential to design and implement effective professional service training programs towards sharpening pharmacists’ knowledge and practical clinical skills to provide consistent, evidence based and integrated healthcare system weight management services. The strong cooperation between the professional associations, faculty of pharmacy, continuing education centers, and practicing pharmacists contributed effectively in the development of community pharmacy services [40]. Attention should also be given in the teaching of undergraduate pharmacy students to ensure that they are equipped with the knowledge and skills needed for the roles of health promotion and disease prevention [41].

Respondents’ beliefs that obese people lack will power and do not adhere to weight loss interventions did not negatively affect their frequency of counseling, which signify that their personal biases have not interfered with their counseling frequency.

The current results identified that lack of pharmacists’ time is one of the main barriers that needs to be overcome. Lack of time is the most significant obstacle standing against the implementation of nontraditional roles of pharmacists worldwide [30,42-44]. Pharmacists could make more time available if there is better delineation between the roles of the pharmacist and the technician. If pharmacists were less involved in dispensing and preparation duties, this would “free-up” time for patient-focused care [45]. It may be cost-effective to employ more pharmacists if this will attract more customers by extended services provided by pharmacists. This is consistent with the fourth and fifth strategies identified by this study to overcome barriers, which are increased staffing in pharmacy and greater support from pharmacy management for counseling activities.

Unlike pharmacists in previous studies [30,46], this study showed that pharmacists did not indicate lack of remuneration as one of the important perceived barriers to obesity counseling. Lack of time as a main barrier in this study may be due to that respondents would want remuneration for something they see as an extra activity since compensation is an issue for many pharmacists in international studies. Pharmacists should be compensated for providing substantial investment in knowledge, skills and time for a service. If payments for counseling were provided, then services would be more likely to be provided, and pharmacists would be more proactive [47].

### Strengths and limitations

The strength of this study is that we used appropriate sampling method and sample size to generate a representative data about the study population. Further strength is the high response rate. The results can, therefore, be generalized at the study population level in Kuwait. On a whole, this study fill in an important gap in literature and provides useful piece of information for obesity counseling by pharmacists in the Middle East. We acknowledge that this type of study, using a self-administered questionnaire, has its limitations. The information given by respondents may be influenced by what is perceived to be the right answer to give. The extent of truthful answers or verifying respondents’ claims is not possible in this type of study. A further limitation of the study is the cross-sectional nature of the data that represented one point in time and, therefore, do not reflect any changes in respondents’ beliefs over time in relation to obesity. A final limitation was that responses of the study participants may have depended on their ability to recall experiences with their patients.

### Future research

This study identified the perceived barriers to obesity counseling by community pharmacists and provided strategies to overcome them. Further research to elucidate in the understanding of barriers and facilitators will help in the adoption and implementation of consistent, evidence based and integrated healthcare system weight management services in the community pharmacies of Kuwait.

## Conclusions

Overall mean responses of counseling obese patients by pharmacists were positively correlated with their perceived comfort with counseling and perceived effectiveness of obesity management aspects. Similarities as well as differences in beliefs and practices were identified between pharmacists in Kuwait and those in developed countries. Due to the high prevalence of overweight and obesity among the population in Kuwait, there is a greater need for pharmacist involvement in helping to tackle this problem, than in many other developed countries where it is already happening.

The involvement of the respondents in obesity counseling, in addition to their declaration of the perceived barriers and strategies to overcome them would certainly be an advantage to the provision of weight management services in community pharmacies of Kuwait. Furthermore, pharmacists who effectively perform obesity counseling need to be identified so that they may act as a role model for others.

## Competing interests

The authors declare that they have no competing interests.

## Authors’ contributions

AA designed and supervised the study, contributed in data collection, performed major parts of the data analysis and wrote the manuscript. MW contributed to the data analysis and interpretation, the discussion and reviewed the manuscript. All authors read and approved the final manuscript.

## Pre-publication history

The pre-publication history for this paper can be accessed here:

http://www.biomedcentral.com/1471-2458/12/863/prepub
